# Diminished Activity of RNA Polymerase III Selectively Disrupts Tissues with the Most Actively Dividing Cells

**DOI:** 10.1371/journal.pbio.0050286

**Published:** 2007-11-27

**Authors:** Lynne Marshall, Sarah J Goodfellow, Robert J White

**Affiliations:** Lynne Marshall and Robert J. White are with the Beatson Institute for Cancer Research, Glasgow, United Kingdom. Sarah J. Goodfellow is with the Faculty of Life Sciences, University of Manchester, Manchester, United Kingdom

The job of transcribing a cell's nuclear genes is shared by three enzymes called RNA polymerases. Each is essential for life, and each transcribes a different set of genetic templates. Focusing on RNA polymerase III (Pol III), which is implicated in both heart disease and cancer, an exciting new paper by Yee et al. supports the growing conviction that Pol III might prove useful as a novel therapeutic target.

Pol III is highly specialized to produce only short untranslated RNAs, such as the transfer RNAs (tRNAs), which convert genetic information into protein sequences. Pol III activity is tightly linked to cell proliferation, probably because of tRNA's role in protein synthesis. To maintain a constant size over successive generations, cells must double their mass—consisting of 80%–90% protein—before they divide. Ill-defined checkpoints ensure that adequate mass has accumulated before the cell enters the S phase, when DNA is replicated. Thus, S phase entry and subsequent cell division depend on a cell's ability to accumulate protein. S phase entry can be prevented in both yeast and cultured rodent cells by mutations in Pol III. Proliferation can be restrained when the p53 and retinoblastoma (RB) tumor suppressors inhibit Pol III transcription. Abnormal Pol III activity is a recurrent feature of human cancers [1].

The Pol III machinery has been very well characterized through genetic and biochemical approaches in yeast and has also been studied in mammalian cell lines. However, it has yet to be subjected to genetic analyses in vertebrates—a deficiency that Yee et al. have begun to redress using zebrafish. They focused on a mutation called *slim jim*, which was recovered in a large-scale mutagenesis screen for altered intestinal morphology. The mutation, which causes short deletions in Rpc2 (one of the 17 Pol III subunits), was predicted to affect the interface between Rpc2 and another subunit, Rpc11. To test this, the authors engineered an equivalent deletion in yeast Rpc2 and showed that the deletion compromises binding of Rpc11. Furthermore, they showed that the *slim jim* defect can be corrected by overexpressing Rpc11 in zebrafish. These elegant experiments provide strong evidence for the predicted interaction between Rpc2 and Rpc11 in a complex metazoan.

Apart from the technical tour de force represented by this work, the consequences of the *slim jim* mutation are fascinating. As expected, the expression of Pol III products is compromised in the mutant, with tRNA levels reduced to about 40% of wild type. What is striking, however, is that the effects of this defect are only apparent in a subset of tissues, at least at the larval stages examined. These were highly proliferative tissues, including the liver and the digestive system. Effects were much less pronounced in tissues composed primarily of quiescent post-mitotic cells, such as those in heart and skeletal muscle. Severe defects were seen in the exocrine pancreas, which is populated with dividing cells, whereas normality reigned in the adjacent pancreatic islets, which stop expanding early in development. Cell cycle analysis suggested that S phase entry was reduced by nearly 5-fold in the exocrine pancreas and 2-fold in the intestinal epithelium. This is strongly reminiscent of the cell-cycle arrest shown to result from certain temperature-sensitive Pol III mutations in budding yeast and cultured rodent cells.

Why do many organs appear normal when all cells depend on protein synthesis and hence tRNA and Pol III transcription for viability? Quiescent cells have much lower demands in this regard and so may cope with the diminished output of the *slim jim* mutant. Mitogenic stimuli trigger a substantial increase in the production of tRNA and protein that is required for passage into S phase; in all probability, the mutant Pol III is unable to meet the increase in demand that is essential for cell replication.

What are the practical implications of Yee and colleagues' evidence that diminished Pol III activity can interfere selectively with the most actively dividing groups of cells in an animal? It provides proof of principle that Pol III transcription offers a viable opportunity for attacking the most proliferative cells in a complex organism. A drug that specifically diminishes Pol III output, perhaps much less drastically than the *slim jim* mutation, might serve to arrest the cell cycle progression of cancer cells without causing excessive damage to healthy tissues with lower protein synthetic demands. As well as in hyperproliferative diseases, elevated Pol III activity is also a feature of cardiac hypertrophy, which depends on high rates of protein synthesis in the absence of cell division [2]. Slim jim might therefore be pointing the way toward novel routes for therapeutic intervention in cardiovascular disease as well as in cancer. Not bad for a skinny fish.
